# Cholesteryl Ester Transfer Protein Inhibition Reduces Major Adverse Cardiovascular Events by Lowering Apolipoprotein B Levels

**DOI:** 10.3390/ijms23169417

**Published:** 2022-08-20

**Authors:** Adam J. Nelson, Allan D. Sniderman, Marc Ditmarsch, Mary R. Dicklin, Stephen J. Nicholls, Michael H. Davidson, John J. P. Kastelein

**Affiliations:** 1 Victorian Heart Institute, Monash University, Clayton, VIC 3800, Australia; 2 Mike and Valeria Rosenbloom Centre for Cardiovascular Prevention, Department of Medicine, McGill University Health Centre, Montreal, QC H4A 3J1, Canada; 3 NewAmsterdam Pharma, 1411 DC Naarden, The Netherlands; 4 Midwest Biomedical Research, Addison, IL 60101, USA; 5 Department of Vascular Medicine, Academic Medical Center, University of Amsterdam, 1105 AZ Amsterdam, The Netherlands

**Keywords:** cholesteryl ester transfer protein, apolipoprotein B, atherosclerotic cardiovascular disease

## Abstract

Cholesteryl ester transfer protein (CETP) facilitates the exchange of cholesteryl esters and triglycerides (TG) between high-density lipoprotein (HDL) particles and TG-rich, apolipoprotein (apo) B-containing particles. Initially, these compounds were developed to raise plasma HDL cholesterol (HDL-C) levels, a mechanism that was previously thought to lower the risk of atherosclerotic cardiovascular disease (ASCVD). More recently, the focus changed and the use of pharmacologic CETP inhibitors to reduce low-density lipoprotein cholesterol (LDL-C), non-HDL-C and apoB concentrations became supported by several lines of evidence from animal models, observational investigations, randomized controlled trials and Mendelian randomization studies. Furthermore, a cardiovascular outcome trial of anacetrapib demonstrated that CETP inhibition significantly reduced the risk of major coronary events in patients with ASCVD in a manner directly proportional to the substantial reduction in LDL-C and apoB. These data have dramatically shifted the attention on CETP away from raising HDL-C instead to lowering apoB-containing lipoproteins, which is relevant since the newest CETP inhibitor, obicetrapib, reduces LDL-C by up to 51% and apoB by up to 30% when taken in combination with a high-intensity statin. An ongoing cardiovascular outcome trial of obicetrapib in patients with ASCVD is expected to provide further evidence of the ability of CETP inhibitors to reduce major adverse cardiovascular events by lowering apoB. The purpose of the present review is to provide an up-to-date understanding of CETP inhibition and its relationship to ASCVD risk reduction.

## 1. Introduction

Cholesteryl ester transfer protein (CETP) is a glycoprotein synthesized in the liver that is present in all primates (including humans), rabbits and hamsters [1]. The CETP molecule has a boomerang shape, with a tunnel that forms between spaces at each end for binding cholesteryl esters and triglycerides (TG) [2]. It is responsible for the exchange of TG from very low-density lipoprotein (VLDL) particles with cholesteryl esters from high-density lipoprotein (HDL) particles and low-density lipoprotein (LDL) particles [3,4,5]. The net effect of this exchange is to enrich VLDL and LDL particles with cholesteryl esters and deplete them of TG while simultaneously enriching HDL particles with TG and depleting them of cholesteryl esters [3,4,5,6].

Epidemiological evidence that elevated HDL cholesterol (HDL-C) is inversely associated with atherosclerotic cardiovascular disease (ASCVD) [7,8,9,10,11], combined with the discovery of mutations in the *CETP* gene that are associated with increased HDL-C and reduced LDL cholesterol (LDL-C) concentrations [12,13], launched interest in the development of agents to pharmacologically inhibit CETP. CETP inhibitors reduce the rate of transfer of cholesteryl ester from HDL into TG-rich lipoproteins. This leads to the formation of larger, cholesteryl ester-enriched HDL particles that are slowly catabolized, and, as a consequence, depletion of cholesterol in the TG-rich and apolipoprotein (apo) B-containing lipoproteins, i.e., VLDL, LDL, intermediate density lipoproteins (IDL), chylomicrons, and their remnants [14].

To date, no CETP inhibitor has ever achieved marketing authorization. The first CETP inhibitors were primarily developed to increase HDL-C, which at the time was believed to be a pathway for ASCVD risk reduction [7,8,9,10,11]. However, randomized controlled trials (RCTs) of agents that primarily raise HDL-C have not shown HDL-C as a causative factor in the reduction of the risk of ASCVD [15]. This includes the cardiovascular outcome trials of the first CETP inhibitors, torcetrapib, dalcetrapib (CETP modulator/functional CETP inhibitor), and evacetrapib, which all failed to reduce ASCVD risk for a variety of agent-specific reasons [3,16,17]. However, the Randomized Evaluation of the Effects of Anacetrapib through Lipid-modification (REVEAL) trial demonstrated a significant reduction in major coronary events with anacetrapib, a potent CETP inhibitor that not only raised HDL-C, but also lowered LDL-C by 17% and apoB by 18%, relative to placebo [18,19]. The absolute LDL-C difference between the anacetrapib and placebo arms amounted to 11 mg/dL (0.28 mmol/L) which places the REVEAL primary endpoint exactly on the Cholesterol Treatment Trialists’ meta-regression line for non-HDL-C and major adverse cardiovascular events (MACE) [20]. The causality of LDL-C and VLDL-C with increased ASCVD risk is supported by a large body of evidence from observational investigations, RCTs, and Mendelian randomization studies, but the relationship loses significance when apoB is accounted for, supporting the view that it is the number of circulating apoB-containing lipoprotein particles that drives risk [21,22,23,24,25].

The purpose of this review is to describe the data supporting the hypothesis that CETP inhibitors have the capacity to reduce MACE as a consequence of their effects on apoB. This is an important concept for the development of new CETP inhibitors, such as obicetrapib, the only CETP inhibitor still in phase 3 clinical development, which has been shown to substantially lower LDL-C and apoB and is being investigated in the ongoing Cardiovascular Outcome Study to Evaluate the Effect of Obicetrapib in Patients with Cardiovascular Disease (PREVAIL) [26].

## 2. Animal Studies of CETP Inhibition

Rodents, which lack CETP, are naturally resistant to the development of atherosclerosis, but in studies wherein the *CETP* gene was introduced (i.e., knock-in transgenic models), plasma HDL-C was decreased, LDL-C increased and atherosclerosis emerged [27,28,29]. Further, in a study of APOE*3-Leiden mice expressing CETP, treatment with anacetrapib dose-dependently reduced atherosclerotic lesion area and increased plaque stability [30]. Non-HDL-C, but not HDL-C, was independently associated with atherosclerotic lesion size.

In rabbits, which have a high plasma CETP concentration and are susceptible to the development of atherosclerosis, CETP inhibition via a variety of strategies including torcetrapib, a CETP antisense oligonucleotide and an anti-CETP vaccine, reduced atherosclerosis [1,31,32,33]. CETP inhibition with dalcetrapib was also shown to reduce atherosclerosis, increase HDL-C, and reduce non-HDL-C in normolipidemic rabbits [34,35], but when administered to cholesterol-fed rabbits with severe hypercholesterolemia, it did not reduce atherosclerosis, despite significantly increasing HDL-C [36]. Furthermore, correlation analysis demonstrated that non-HDL-C and TG levels had a direct relationship with the development of atherosclerosis, but HDL-C levels again did not [36].

## 3. Observational and Mendelian Randomization Studies

In observational investigations in humans, certain *CETP* gene polymorphisms have been shown to be associated with decreased CETP activity, increased HDL-C and decreased LDL-C concentrations, as well as reduced risk of ASCVD [12,13,22,37,38,39,40,41]. The Copenhagen City Heart Study, which followed more than 10,000 participants for up to 34 years, found that two common *CETP* gene polymorphisms which reduce CETP activity were associated with significant reductions in the risks for any ischemic vascular event (hazard ratio [HR] 0.76; 95% confidence interval [CI] 0.68–0.85), ischemic heart disease, myocardial infarction, ischemic cerebrovascular disease, ischemic stroke, and total mortality, as well as an antiatherogenic lipid profile (increased HDL-C and decreased LDL-C, non-HDL-C, and TG) [38]. A meta-analysis by Nomura et al. of nearly 60,000 participants from 12 case-control studies found that, compared with non-carriers, carriers of CETP truncating variants had significantly higher HDL-C (22.6 mg/dL [0.58 mmol/L]), lower LDL-C (−12.2 mg/dL [−0.32 mmol/L]) and TG (−6.3%), and reduced risk of coronary heart disease (CHD) (summary odds ratio 0.70; 95% CI 0.54–0.90) [40]. The magnitude of the effect on CHD risk was consistent with the magnitude of LDL-C lowering associated with CETP-inhibiting variants.

A Mendelian randomization analysis of individual-participant data from over 100,000 participants in 14 cohort and case-control studies (with external validation in nearly 190,000 participants from 48 additional studies) conducted by Ference et al. examined the association between changes in LDL-C (and other lipoproteins) and the risk of cardiovascular events related to variants in the *CETP* gene [22]. A CETP genetic score was calculated by summing the number of HDL-C-raising alleles that each participant inherited at each variant thereby simulating the effect of CETP inhibition (high scores, high HDL-C). The same approach was used to simulate the effect of statins by generating a 3-hydroxy-3-methylglutaryl-coenzyme A reductase (HMGCR) genetic score based on LDL-C lowering alleles (high scores, low LDL-C). Participants with high CETP scores were then dichotomized by high and low HMGCR gene scores, thereby modelling the impact of CETP inhibitors with or without statin background [22,42]. Modelling CETP monotherapy, higher CETP genetic scores were associated with higher HDL-C, lower LDL-C, lower apoB, and a corresponding lower risk of major vascular events (odds ratio 0.946; 95% CI 0.921–0.972). Of note, the risk reduction per unit change in LDL-C and apoB levels was of the same magnitude as found for genetic variants associated with other targets of LDL-C-lowering therapies with proven MACE reduction, including *HMGCR*, Nieman-Pick C1 Like-1 (*NPC1L1*) and proprotein convertase subtilisin kexin type 9 (*PCSK9*) [22,42]. Furthermore, the external validation analysis showed that a genetic score that consisted of variants associated with discordant changes between LDL-C and apoB concentration, similar to that seen when *CETP* variants are combined with *HMGCR* variants, was associated with a similar risk of CHD per unit change in apoB, but an attenuated risk of CHD per unit change in LDL-C. This led the investigators to conclude that the clinical benefit on MACE of lowering LDL-C levels may depend on the corresponding reduction in apoB-containing lipoprotein particles, rather than the reduction in cholesterol carried by LDL particles. Thus, the accumulating evidence from research in animal models, observational investigations, and Mendelian randomization studies points to the effects of CETP on apoB, rather than effects on HDL-C (or LDL-C), as the predominant mechanism for reducing risk of ASCVD. The concept of apoB as the most important determinant of ASCVD risk is described in more detail later in this review.

## 4. Cardiovascular Outcome Trials of CETP Inhibitors

Clinical outcome trials of four CETP inhibitors have been completed, including for torcetrapib, dalcetrapib, evacetrapib and anacetrapib. The most recent CETP inhibitor in clinical development, obicetrapib, is undergoing investigation in PREVAIL, a cardiovascular outcome trial which is expected to be completed in 2026 [26]. Changes in LDL-C, apoB, and HDL-C levels and results for the coronary or cardiovascular endpoints from large trials of each of the CETP inhibitors are summarized in Table 1. Torcetrapib, dalcetrapib, and evacetrapib all failed to demonstrate significant ASCVD risk reduction [3,16,17]. These failures were determined to be related to compound-specific factors and were not target related, as described in more detail below. However, the REVEAL trial of anacetrapib demonstrated a significant reduction in major coronary events [18].

REVEAL was a randomized, double-blind, placebo-controlled trial that administered 100 mg/d anacetrapib vs. placebo to 30,449 men and women with ASCVD receiving intensive atorvastatin therapy and with mean LDL-C of 61 mg/dL (1.58 mmol/L), mean non-HDL-C of 92 mg/dL (2.38 mmol/L), and mean HDL-C of 40 mg/dL (1.03 mmol/L). Subjects were followed for a median of 4.1 years. The primary outcome was a first major coronary event, defined as a composite of coronary death, myocardial infarction, or coronary revascularization, which occurred in significantly fewer patients in the anacetrapib group (10.8%) than in the placebo group (11.8%) (rate ratio 0.91; 95% CI 0.85–0.97; *p* = 0.004) [18].

**Figure 1:**
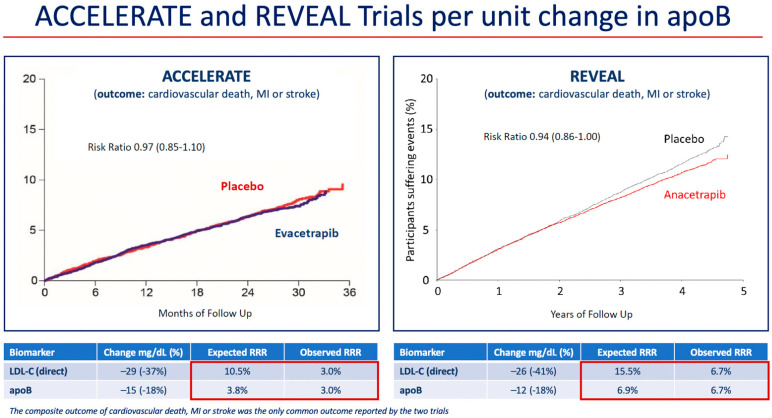
Reduction in risk of coronary or cardiovascular events per unit change in apoB in the cardiovascular outcome trials of evacetrapib and anacetrapib. Red boxes mark the expected and observed RRR associated with LDL-C and apoB changes in each trial. Abbreviations: apo, apolipoprotein; ACCELERATE, Assessment of Clinical Effects of Cholesteryl Ester Transfer Protein Inhibition with Evacetrapib in Patients at a High Risk for Vascular Outcomes; LDL-C, low-density lipoprotein cholesterol; MI, myocardial infarction; REVEAL, Randomized Evaluation of the Effects of Anacetrapib through Lipid-modification; RRR, relative risk reduction.

At 2 years, mean HDL-C was 43 mg/dL (1.12 mmol/L) higher in the anacetrapib group compared with the placebo group (relative difference of 104%), and mean LDL-C measured by a direct assay was 26 mg/dL (0.68 mmol/L) lower in the anacetrapib group compared with the placebo group (relative difference of −41%). However, because the direct assay was shown to underestimate the LDL-C concentration in patients treated with anacetrapib in Determining the Efficacy and Tolerability of CETP Inhibition with Anacetrapib (DEFINE) trial, a subgroup analysis of LDL-C using beta-quantification, also known as preparative ultracentrifugation, which is the gold standard measurement, was also conducted in 2000 subjects in REVEAL [44,45]. Using beta-quantification, the absolute difference in LDL-C between treatments was 11 mg/dL (0.3 mmol/L) (relative difference of −17%). These results were consistent with those for the mean apoB concentration, which was 12 mg/dL lower in the anacetrapib group compared to the placebo group (relative difference of 18%). An examination of the risk reduction differences in the ACCELERATE and REVEAL trials demonstrated agreement between the predicted and observed differences in event incidence based on differences in apoB concentration (Figure 1) [18]. Furthermore, the interventions in REVEAL and in the Improved Reduction of Outcomes: Vytorin Efficacy International Trial (IMPROVE-IT) (anacetrapib and ezetimibe, respectively) reduced apoB by approximately the same amount, and this translated into similar reductions in risk of MACE (defined as the composite of cardiovascular death, myocardial infarction, ischemic stroke, or coronary revascularization) (Table 2) [18,46].

Anacetrapib is highly lipophilic and accumulates in adipose tissue [47,48]. Consequently, its effects on lipids can be observed for years after completing treatment [48,49]. An investigation of efficacy and safety in 26,129 subjects during an additional 2.3 years median follow-up after the end of the treatment period in REVEAL was conducted [49]. It showed that at the final follow-up visit, mean HDL-C was 44 mg/dL (1.13 mmol/L) higher and non-HDL-C was 17 mg/dL (0.44 mmol/L) lower in subjects who had been allocated to anacetrapib [49]. Furthermore, there was a 20% additional significant reduction in major coronary events compared to the initial trial endpoint (*p* < 0.001). Overall, during the combined median follow-up period of 6.3 years, there was a 12% (95% CI 7–17%) proportional reduction in major coronary events, corresponding to a 1.8% (95% CI 1.0–2.6%) absolute reduction. Despite overall favorable results from REVEAL, the magnitude of effect on coronary events was relatively modest and given the persistent concern about accumulation in adipose tissue, Merck did not pursue regulatory approval for anacetrapib [50].

REVEAL was the first RCT to demonstrate that adding CETP inhibitor therapy to intensive statin therapy reduced the risk of coronary events. All previous cardiovascular outcome trials had failed to support this hypothesis. The Investigation of Lipid Level Management to Understand its Impact in Atherosclerotic Events (ILLUMINATE) trial of torcetrapib, the first CETP inhibitor to be developed, was terminated early because of an increased incidence of cardiovascular events (HR 1.25; 95% CI 1.09–1.44; *p* = 0.001) and all-cause mortality (HR 1.58; 95% CI 1.14–2.19; *p* = 0.006) with torcetrapib compared with placebo, despite increasing HDL-C by 72.1% and decreasing LDL-C by 24.9% [3]. The increased risk has since been shown to be related to off-target, agent-specific effects of torcetrapib including electrolyte abnormalities and increased systolic blood pressure, aldosterone, cortisol and endothelin-I [3,51,52,53]. Other CETP inhibitors that have been developed since torcetrapib have not been associated with any notable off-target effects [6,43,54,55,56,57].

The Randomized, Double-blind, Placebo-controlled Study Assessing the Effect of RO4607381 on Cardiovascular Mortality and Morbidity in Clinically Stable Patients with a Recent Acute Coronary Syndrome (Dal-OUTCOMES) of dalcetrapib was terminated early for futility after a pre-specified interim analysis when there was 71% of the projected total number of primary endpoint events after a median follow-up of 31 months. It showed no effect on the primary endpoint (HR 1.04; 95% CI 0.93–1.16; *p* = 0.52) [16]. Dalcetrapib increased HDL-C levels from baseline by 31–40% in the dalcetrapib group compared with 4–11% in the placebo group but had minimal effects on LDL-C and apoB concentrations.

**Figure 2:**
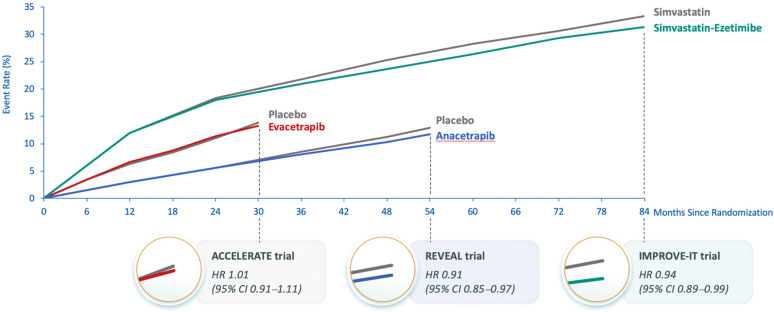
Kaplan–Meier curves from cardiovascular outcome trials of anacetrapib, evacetrapib and ezetimibe. Abbreviations: ACCELERATE, Assessment of Clinical Effects of Cholesteryl Ester Transfer Protein Inhibition with Evacetrapib in Patients at a High Risk for Vascular Outcomes; CI, confidence interval; HR, hazard ratio; IMPROVE-IT, Improved Reduction of Outcomes: Vytorin Efficacy International Trial; REVEAL, Randomized Evaluation of the Effects of Anacetrapib through Lipid-modification.

The Assessment of Clinical Effects of Cholesteryl Ester Transfer Protein Inhibition with Evacetrapib in Patients at a High-Risk for Vascular Outcomes (ACCELERATE) trial was also terminated early due to futility when there was 82% of the planned primary endpoints after a median follow-up of 26 months. It showed no effect on the primary endpoint (HR 1.01; 95% CI 0.91–1.11; *p* = 0.91) [17]. At 3 months of treatment, evacetrapib increased HDL-C by 133% vs. 1.6% with placebo, and using a direct assay, LDL-C levels were reduced by 31.1% with evacetrapib vs. a 6.0% increase with placebo. However, the apoB results did not concur with the LDL-C results; apoB was reduced by 15.5% with evacetrapib and increased by 3.8% with placebo. This suggested that, similar to anacetrapib, the direct assay might overestimate LDL-C reduction with evacetrapib. The observed MACE in ACCELERATE was similar to that expected based on the apoB reduction (Figure 1) [17]. A likely explanation for the failure to demonstrate significant MACE reduction in ACCELERATE is the short duration of treatment [14]. In IMPROVE-IT, ezetimibe reduced apoB by approximately the same amount as did evacetrapib in ACCELERATE, but the Kaplan–Meier curves for the primary composite MACE endpoint in IMPROVE-IT did not show a separation between ezetimibe and placebo until approximately 36 months of treatment [46], which was longer than the 26 months duration of ACCELERATE (Figure 2). The Kaplan–Meier curves for the primary composite coronary event endpoint in REVEAL show a similar delay in separation between anacetrapib and placebo until approximately 36 months of treatment. This comparison demonstrates the importance of both apoB reduction and duration of treatment. The reduction in risk is also correlated with the duration of treatment, regardless of how LDL-C (and apoB) is reduced, and clinically relevant risk reduction due to lipid lowering often does not emerge until after approximately 2 years [57].

## 5. Obicetrapib—The Newest CETP Inhibitor

Obicetrapib (also known as TA-8995) is a new selective CETP inhibitor designed specifically to reduce LDL-C and apoB [43,56,58]. It is the most polar of all CETP inhibitors, and its biochemical structure is suggested to have improved binding and specificity [58,59]. It has been shown to inhibit the activity of CETP by up to 97% [58]. Obicetrapib was shown to have a mean terminal half-life at steady-state of ~130–150 h after 5 and 10 mg repeated daily dosing [58]. The CETP Inhibition by Obicetrapib in Patients with Mild Dyslipidemia (TULIP) phase 2 trial evaluated obicetrapib at several doses alone and in combination with moderate-intensity statins in 364 participants with mild dyslipidemia (LDL-C > 2.5 mmol/L [96.7 mg/dL] and <4.5 mmol/L [173 mg/dL], HDL-C < 1.8 mmol/L [69.6 mg/dL] and >0.8 mmol/L [30.9 mg/dL], and TG < 4.5 mmol/L [399 mg/dL]) [56]. Subjects received placebo or obicetrapib at doses of 1, 2.5, 5, or 10 mg alone and obicetrapib 10 mg with atorvastatin 20 mg or rosuvastatin 10 mg once daily for 12 weeks [56]. LDL-C measured by beta-quantification was significantly reduced by 27.4%, 32.7%, 45.3%, and 45.3% with the 1, 2.5, 5, and 10 mg obicetrapib doses, respectively, and by 68.2% and 63.8% with obicetrapib plus atorvastatin and plus rosuvastatin, respectively. Similarly, apoB was significantly reduced by 20.0%, 24.6%, 33.6%, and 33.7% with the 1, 2.5, 5, and 10 mg obicetrapib doses, respectively, and by 50.1% and 46.3% with obicetrapib plus atorvastatin and plus rosuvastatin, respectively. Obicetrapib also significantly reduced non-HDL-C and lipoprotein(a) and increased HDL-C. In contrast to anacetrapib, obicetrapib is more hydrophilic and was undetectable in plasma within 120 h after discontinuation of dosing.

The Randomized Study of Obicetrapib as an Adjunct to Statin Therapy (ROSE) administered obicetrapib 5 mg and 10 mg once daily compared with placebo as an adjunct to high-intensity statin therapy (atorvastatin 40 or 80 mg or rosuvastatin 20 or 40 mg) for an 8-week treatment period to 120 subjects with LDL-C > 70 mg/dL (1.81 mmol/L) and TG < 400 mg/dL (4.52 mmol/L) [43]. In ROSE, the primary LDL-C analysis was performed using the Friedewald formula to calculate LDL-C, which demonstrated median LDL-C reductions from baseline of 42.9% and 45.7% for the obicetrapib 5 mg and 10 mg groups, respectively, compared with 0.0% for placebo. LDL-C results using beta-quantification were comparable to those from Friedewald, −41.5% and −50.8% for 5 mg and 10 mg obicetrapib, respectively, compared with −6.50% with placebo. ApoB was significantly reduced by 24.4% and 29.8%, non-HDL-C by 38.9% and 44.4%, and lipoprotein(a) by 33.8% and 56.5%, respectively, and HDL-C was increased by 135% and 165% and apoA1 by 44.6% and 47.8%, respectively, for 5 mg and 10 mg obicetrapib (*p* < 0.0001 compared with placebo). To date, obicetrapib has consistently been shown to be safe and well tolerated at all doses alone and in combination with statins [43,56,58]. The PREVAIL cardiovascular outcome trial is administering 10 mg obicetrapib vs. placebo to 9000 participants with ASCVD and LDL-C ≥ 70 mg/dL (1.80 mmol/L) and TG < 400 mg/dL (4.52 mmol/L), despite taking maximal tolerated lipid-lowering therapy [26]. Because obicetrapib reduced LDL-C and apoB in ROSE by approximately three times the level shown for anacetrapib in REVEAL, it could potentially result in a greater reduction in ASCVD risk.

## 6. Modulation of ApoB as the Basis for CETP Inhibitor Reduction of Cardiovascular Events

As described previously, CETP inhibition reduces the rate of transfer of cholesteryl ester from HDL into TG-rich lipoproteins, thereby increasing the overall cholesterol content in HDL and the formation of larger HDL particles that are more slowly catabolized, while also depleting the cholesterol content of the apoB lipoproteins, including VLDL, LDL, chylomicrons and their remnants [14]. CETP inhibitors also improve cholesterol efflux capacity, the first step in reverse cholesterol transport [17,56,60], and reduce lipoprotein(a) [43,56,61]. During the initial development of CETP inhibitors, their action on HDL-C was the primary focus. However, the focus of CETP inhibitors has now shifted to their ability to reduce LDL-C and apoB [62]. An examination of apoB kinetics in 19 subjects with dyslipidemia who received 120 mg torcetrapib with or without atorvastatin for 4 weeks demonstrated that torcetrapib reduced VLDL, IDL, LDL and apo B levels primarily by increasing the rate of apoB100 clearance, whereas, when added to atorvastatin, torcetrapib reduced apoB levels mainly by enhancing VLDL apoB100 clearance and reducing production of IDL and LDL apoB100 [63]. A study of apoB kinetics in 39 mildly hypercholesterolemic subjects who received 100 mg anacetrapib added to their background treatment of atorvastatin for 8 weeks demonstrated that anacetrapib reduced LDL-C levels by increasing the LDL-apoB100 fractional clearance, both when anacetrapib was given alone or on background statin treatment [64]. This suggests a common mechanism of action of anacetrapib and statins to enhance LDL-apoB clearance, thereby reducing the total number of LDL particles as well as LDL-C and apoB concentrations [14,64].

There is universal agreement, supported by prospective observational investigations, RCTs, and Mendelian randomization studies that LDL-C, non-HDL-C, and apoB are causal factors for ASCVD [21,65]. In conventional statistical tests, the three markers are treated as independent variables. However, they are not metabolically and biologically independent. On the contrary, they are metabolically and biologically tightly related, thereby underpinning their statistical correlations. However, they are not identical because the mass of cholesterol within apoB particles is variable. When apoB particles contain more cholesterol than average, LDL-C and non-HDL-C overestimate the number of apoB particles, whereas, when apoB particles contain less cholesterol than average, LDL-C and non-HDL-C underestimate the number of apoB particles. Because there is one molecule of apoB per apoB particle, plasma apoB equals the total number of atherogenic particles in plasma, the great majority of which, generally 90% or more, are LDL particles.

LDL-C remains the most common lipid measure in clinical care, notwithstanding all the evidence that both non-HDL-C and apoB are more accurate markers of cardiovascular risk and acknowledged to be so by the major European and American Guidelines [24,25]. Their superiority over LDL-C is axiomatic since both incorporate VLDL as well as LDL and both VLDL and LDL particles are atherogenic. The real contest is between non-HDL-C and apoB. Multiple, well-conducted, prospective observational studies and randomized clinical trials have shown non-HDL-C and apoB to have similar predictive power, while many have shown apoB is superior to non-HDL-C [66]. One meta-analysis of randomized clinical trials showed non-HDL-C to be statistically, but not clinically, superior to apoB [67] whereas another showed that apoB was superior to non-HDL-C [68]. This mixed picture of results reflects the fact that the conventional statistical methods were not designed to compare highly correlated variables such as non-HDL-C and apoB.

Discordance analysis was created to overcome this limitation [69]. Because the cholesterol content of apoB particles is variable, discordant groups can be created in which the non-HDL-C/apoB ratio is either high or low. In both cases, non-HDL-C and apoB will make opposite predictions about risk. Discordance analyses in the Framingham Heart Study [70], INTERHEART [71], the Women’s Health Study [72] and UK Biobank [73] have all shown that apoB, not HDL-C, correctly predicts cardiovascular risk when the two markers are discordant. Similarly, discordance analysis in the Coronary Artery Risk Development in Young Adults (CARDIA) study has shown apoB correctly predicts the risk of coronary artery calcification whereas non-HDL-C does not [74]. Moreover, Johannesen et al. in a discordance analysis of subjects on statin therapy demonstrated that apoB was superior to non-HDL-C as a marker of cardiovascular risk overall and cardiovascular mortality specifically [75].

The uniform outcome in favor of apoB in discordance analyses establishes, therefore, that when LDL-C or non-HDL-C differ in prediction of cardiovascular risk from apoB, apoB is right and LDL-C or non-HDL-C is wrong. Moreover, clinically significant discordance is common. Framingham [70], INTERHEART [71], and the Women’s Health Study [72] all demonstrated that significant discordance between LDL-C and apoB and between non-HDL-C and apoB is present in up to two-thirds of the population. In individuals with significant discordance, apoB and the cholesterol markers will make different predictions as to risk even if the HRs for the whole population are the same [76]. This follows because a HR is the increase in risk per standard deviation increase in the risk of the marker. Thus, if the level of apoB in an individual, relative to the population, is higher than the level of LDL-C or non-HDL-C, the hazard predicted in this patient will be higher than the hazard predicted by LDL-C or non-HDL-C, even if the HRs calculated for the population are the same. Thus, even in the studies in which the overall HRs for non-HDL-C, LDL-C and apoB were the same, such as the Emerging Risk Factor Collaboration [11], apoB will offer significant advantages to a substantial number of subjects.

Strong evidence in favor of apoB is now available from multiple Mendelian randomization analyses. A de novo genome-wide association study was conducted to examine lipid-related traits (apoB, LDL-C, TG, HDL-C, and apoA1) associated with risk of CHD using data from the UK Biobank (~440,000 participants) followed by Mendelian randomization analysis using data from the CARDIoGRAMplusC4D consortium with over 60,000 cases of CHD [77]. Mendelian randomization indicated that LDL-C, TG, and apoB were associated with increased risk of CHD, and that HDL-C and apoA1 were associated with decreased CHD risk. However, in multivariable Mendelian randomization, only apoB was robustly related to increased CHD risk (odds ratio 1.92; 95% CI 1.31–2.81), whereas the relationships for each of the other lipid-related traits when included with apoB in the model were either attenuated to the null or changed direction. The primacy of apoB as a risk determinant for ASCVD was confirmed in another Mendelian randomization analysis, which also demonstrated that apoB was the most significant lipid-related causal factor for peripheral arterial disease [78].

Recently, a prospective cohort analysis of data from the UK Biobank and from two large clinical trials, Further Cardiovascular Outcomes Research with PCSK9 Inhibition in Subjects with Elevated Risk (FOURIER) and IMPROVE-IT, was undertaken to examine whether the cholesterol content or the TG content of lipoproteins is associated with ASCVD risk beyond the number of apoB-containing lipoproteins [79]. Among nearly 400,000 primary prevention individuals and over 40,000 patients with established ASCVD, apoB, non-HDL-C, and TG were each individually shown to be associated with incident myocardial infarction, but, when assessed together, only apoB was significantly associated with increased risk (HR per 1 standard deviation 1.27; 95% CI 1.15–1.40; *p* < 0.001 for the primary prevention analysis). Finally, apoB has now been shown to be a more accurate marker of atherosclerotic cerebrovascular disease than non-HDL-C or LDL-C [80]. None of this contradicts the masses of evidence linking LDL-C and non-HDL-C to the risk of cardiovascular disease. ApoB is simply a more complete measure of the cardiovascular risk associated with the apoB lipoproteins than LDL-C and non-HDL-C. 

## 7. Conclusions

In conclusion, a large body of evidence from animal models, observational cohorts, Mendelian randomization studies and RCTs shows that CETP inhibition reduces LDL-C and apoB and increases HDL-C. Initially developed as a therapeutic class that would favorably modulate atherosclerotic risk through protective increases in HDL-C, emerging evidence suggests it may be through reductions in atherogenic lipoproteins that CETP inhibitors ultimately demonstrate their clinical value. To this end, early phase studies of the newest CETP inhibitor, obicetrapib, reveal the most potent LDL-C and apoB lowering in the class to date, alone or in combination with high-intensity statins. Currently undergoing investigation in a large Phase 3 program, if shown to be both safe and effective, obicetrapib may become the first clinically available CETP inhibitor. Given that the majority of patients do not achieve treatment goals with currently available lipid-lowering therapies, obicetrapib may offer an important adjunct for a large number of patients.

## Figures and Tables

**Table 1:** Lipoprotein lipid, apolipoprotein B, and cardiovascular outcome results from trials with cholesteryl ester transfer protein inhibitors.

Clinical Trial Name	Agent, Daily Dose	Median Follow-Up	Baseline (mg/dL) * and Approximate Relative Difference from Placebo (% Δ)			HR ^†^ (95% CI)
			LDL-C	apoB	HDL-C	
**ILLUMINATE** [3]	Torcetrapib, 60 mg	1.5 y	BL = 80 −28%	BL = 73 −15%	BL = 49 +70%	1.25 (1.09, 1.44)
**Dal-OUTCOMES** [16]	Dalcetrapib, 600 mg	2.6 y	BL = 76 Minimal	BL = 81 Minimal	BL = 42 +29%	1.04 (0.93, 1.16)
**ACCELERATE** [17]	Evacetrapib, 130 mg	2.2 y	BL = 81 −37%	BL = 78 −19%	BL = 45 +132%	1.01 (0.91, 1.11)
**REVEAL** [18]	Anacetrapib, 100 mg	4.1 y	BL = 61 −17% ^‡^	BL NR −18%	BL = 40 +104%	0.91 (0.85, 0.97)
**ROSE ^§^** [43]	Obicetrapib, 5 mg	0.15 y	BL = 95 −35% ^‡^	BL = 88 −22%	BL = 47 +140%	NA
	Obicetrapib, 10 mg	0.15 y	BL = 88 −44% ^‡^	BL = 82 −27%	BL = 44 +170%	NA

* To convert mg/dL to mmol/L for LDL-C and HDL-C, multiply by 0.02586. ^†^ HR is for the primary outcome, which for ILLUMIMATE was defined as death from coronary heart disease, nonfatal myocardial infarction, stroke, or hospitalization for unstable angina; for Dal-OUTCOMES it was defined as death from coronary heart disease, nonfatal myocardial infarction, ischemic stroke, unstable angina, or cardiac arrest with resuscitation; for ACCELERATE it was defined as death from cardiovascular causes, myocardial infarction, stroke, coronary revascularization, or hospitalization for unstable angina; and for REVEAL it was defined as coronary death, myocardial infarction, or coronary revascularization. ^‡^ Result shown is from beta-quantification. ^§^ ROSE was an 8-week, phase 2 dose-finding trial; the Cardiovascular Outcome Study to Evaluate the Effect of Obicetrapib in Patients with Cardiovascular Disease (PREVAIL) is ongoing [26]. Abbreviations: ACCELERATE, Assessment of Clinical Effects of Cholesteryl Ester Transfer Protein Inhibition with Evacetrapib in Patients at a High Risk for Vascular Outcomes; apo, apolipoprotein; BL, baseline; Dal-OUTCOMES, Randomized, Double-blind, Placebo-controlled Study Assessing the Effect of RO4607381 on Cardiovascular Mortality and Morbidity in Clinically Stable Patients with a Recent Acute Coronary Syndrome; CI, confidence interval; HDL-C, high-density lipoprotein cholesterol; HR, hazard ratio; ILLUMINATE, Investigation of Lipid Level Management to Understand its Impact in Atherosclerotic Events; LDL-C, low-density lipoprotein cholesterol; NA, not applicable; NR, not reported; REVEAL, Randomized Evaluation of the Effects of Anacetrapib through Lipid-modification; ROSE, Randomized Study of Obicetrapib as an Adjunct to Statin Therapy.

**Table 2:** Reduction in risk of major cardiovascular events per unit change in apolipoprotein B in cardiovascular outcome trials of anacetrapib and ezetimibe.

Trial Name	No. Participants		No. Events		Median Follow-Up, y	ApoB, Diff. between Treatments, mg/dL *	RR (95% CI) ^†^
	Active	Placebo	Active	Placebo			
**REVEAL** [18]	15,225	15,224	2068	2214	4.6	12	0.934 (0.88, 0.99)
**IMPROVE-IT** [46]	9067	9077	2498	2685	6.1	12	0.931 (0.89, 0.98)
**Combined**	24,292	24,301	4566	4899	5.1	12	0.932 (0.90, 0.97)

* Absolute difference is the on-treatment value in the active group minus the value in the placebo group. ^†^ Major cardiovascular events defined as the composite of cardiovascular death, myocardial infarction, ischemic stroke, or coronary revascularization. Abbreviations: Apo, apolipoprotein; CI, confidence interval; Diff, difference; IMPROVE-IT, Improved Reduction of Outcomes: Vytorin Efficacy International Trial; No, number; REVEAL, Randomized Evaluation of the Effects of Anacetrapib through Lipid-modification; RR, relative risk.
